# Hadron Therapy, Magnetic Nanoparticles and Hyperthermia: A Promising Combined Tool for Pancreatic Cancer Treatment

**DOI:** 10.3390/nano10101919

**Published:** 2020-09-25

**Authors:** Francesca Brero, Martin Albino, Antonio Antoccia, Paolo Arosio, Matteo Avolio, Francesco Berardinelli, Daniela Bettega, Paola Calzolari, Mario Ciocca, Maurizio Corti, Angelica Facoetti, Salvatore Gallo, Flavia Groppi, Andrea Guerrini, Claudia Innocenti, Cristina Lenardi, Silvia Locarno, Simone Manenti, Renato Marchesini, Manuel Mariani, Francesco Orsini, Emanuele Pignoli, Claudio Sangregorio, Ivan Veronese, Alessandro Lascialfari

**Affiliations:** 1 Dipartimento di Fisica and INFN, Università degli Studi di Pavia, 27100 Pavia, Italy; matteo.avolio01@universitadipavia.it (M.A.); maurizio.corti@unipv.it (M.C.); manuel.mariani@unipv.it (M.M.); 2 Dipartimento di Chimica, Università di Firenze and INSTM, 50019 Sesto Fiorentino (FI), Italy; martin.albino@unifi.it (M.A.); andrea.guerrini@sns.it (A.G.); claudia.innocenti@unifi.it (C.I.); claudio.sangregorio@iccom.cnr.it (C.S.); 3 Dipartimento di Scienze and INFN, Università Roma Tre, 00146 Roma, Italy; antonio.antoccia@uniroma3.it (A.A.); francesco.berardinelli@uniroma3.it (F.B.); 4 Dipartimento di Fisica and INFN, Università degli Studi di Milano, 20133 Milano, Italy; paolo.arosio@unimi.it (P.A.); daniela.bettega@mi.infn.it (D.B.); paola.calzolari@unimi.it (P.C.); salvatore.gallo@unimi.it (S.G.); cristina.lenardi@mi.infn.it (C.L.); silvia.locarno@unimi.it (S.L.); renato.marchesini@alice.it (R.M.); francesco.orsini@unimi.it (F.O.); ivan.veronese@unimi.it (I.V.); 5 Fondazione CNAO, 27100 Pavia, Italy; mario.ciocca@cnao.it (M.C.); angelica.facoetti@cnao.it (A.F.); 6 Dipartimento di Fisica, Università degli Studi di Milano and INFN, Lab. LASA, 20090 Segrate (MI), Italy; flavia.groppi@mi.infn.it (F.G.); simone.manenti@mi.infn.it (S.M.); 7 ICCOM-CNR, 50019 Sesto Fiorentino (FI), Italy; 8 C.I.Ma.I.Na., Centro Interdisciplinare Materiali e Interfacce Nanostrutturati, 20133 Milano, Italy; 9 Fondazione IRCSS Istituto Nazionale dei tumori, 20133 Milano, Italy; emanuele.pignoli@istitutotumori.mi.it; 10 INFN, Sezione di Firenze, 50019 Sesto Fiorentino (FI), Italy

**Keywords:** hadron therapy, magnetic nanoparticles, hyperthermia, nanomaterials, magnetic fluid hyperthermia, pancreatic cancer

## Abstract

A combination of carbon ions/photons irradiation and hyperthermia as a novel therapeutic approach for the in-vitro treatment of pancreatic cancer BxPC3 cells is presented. The radiation doses used are 0–2 Gy for carbon ions and 0–7 Gy for 6 MV photons. Hyperthermia is realized via a standard heating bath, assisted by magnetic fluid hyperthermia (MFH) that utilizes magnetic nanoparticles (MNPs) exposed to an alternating magnetic field of amplitude 19.5 mTesla and frequency 109.8 kHz. Starting from 37 °C, the temperature is gradually increased and the sample is kept at 42 °C for 30 min. For MFH, MNPs with a mean diameter of 19 nm and specific absorption rate of 110 ± 30 W/g_Fe3_o_4_ coated with a biocompatible ligand to ensure stability in physiological media are used. Irradiation diminishes the clonogenic survival at an extent that depends on the radiation type, and its decrease is amplified both by the MNPs cellular uptake and the hyperthermia protocol. Significant increases in DNA double-strand breaks at 6 h are observed in samples exposed to MNP uptake, treated with 0.75 Gy carbon-ion irradiation and hyperthermia. The proposed experimental protocol, based on the combination of hadron irradiation and hyperthermia, represents a first step towards an innovative clinical option for pancreatic cancer.

## 1. Introduction

Pancreatic cancer is the seventh leading cause of cancer deaths [1], being responsible for 6% of all cancer-related deaths. Researchers are tirelessly endeavoring to develop therapies for pancreatic adenocarcinoma, but in spite of their efforts, survival rate remains poor and most patients have an unresectable tumor at the time of the diagnosis [2]. To overcome these limits, conventional radiotherapy, commonly carried out with X-ray beams has been applied in the context of neoadjuvant or adjuvant therapy concepts, but only modest results have been obtained both because pancreatic cancer is radioresistant and because of the radiosensitivity of normal tissues and organs surrounding the tumor [3,4,5,6]. Therefore, highly and inherently conformal radiation therapy techniques, e.g., hadron therapy (HT), present promising alternative treatment options. As an emerging approach, the combination of these new modalities with more conventional therapeutical protocols seems to offer a more efficient way to kill cancer tissues and/or to control or possibly inhibit the tumor progression [7,8,9].

Hadron therapy employs radiation beams consisting of charged particles, like protons, carbon and helium ions. HT offers some important advantages in comparison to X-ray radiotherapy: (i) the damage induced on the tumor tissues is generally higher; (ii) as a consequence of point (i), it allows the treatment of radioresistant tumors; (iii) the surrounding healthy cells are kept safe because most energy is deposited within the tumor site (Bragg peak), maximizing the cancer cells damage and (iv) the particle beam remains more collimated along the full path and, therefore, any side effects to the adjacent normal tissues can be further reduced [10].

Among therapies additional to irradiation, hyperthermia (Hyp) is clinically investigated for its efficacy [11,12,13,14,15]. The main reason is that the cancer cells are intrinsically sensitive to hyperthermia, because of the highly disorganized development of the tumor and the consequent blood perfusion distortion, which leads to low pH and hypoxia; such an environment may favor cell death by temperature increase [16,17]. On the contrary, healthy tissues are kept safe because they are rarely in conditions of hypoxia and high acidity. The local heating of tumor tissue has been historically realized in different ways, e.g., bath heating [18], microwave irradiation [19], radiofrequency waves [20], focused ultrasounds [21,22], capacitance hyperthermia [23], concentrated laser light [24], magnetic fluid hyperthermia (MFH) [25,26,27,28,29,30,31,32,33,34] and, more recently, innovative techniques resulting from the coupling of different types of hyperthermia (for example, magnetic and ultrasonic hyperthermia or magnetic hyperthermia and phototherapy [35,36]). In a relatively recent literature-based review, Peeken et al. [37] show the currently used hyperthermia techniques for heat delivery and temperature control, remarking the different modes of action of Hyp. From a different point of view, Datta et al. [38] reviewed the advantages of using multifunctional MNPs in local tumor MFH, and discussed their role as multimodal theranostic vectors.

MFH is used for thermoablation (T > 50 °C) or as mild hyperthermia (40–45 °C) and has a minimal invasivity. It is performed by injecting superparamagnetic nanoparticles directly into the tumor and subsequently exposing the patient to an alternating magnetic field (AMF) of a specific intensity H and frequency *f*. The Brezovich criterion [39] H·*f* < 4.85 × 10^8^ Am^−1^s^−1^, although under discussion [23], is used to save patients’ health. MFH has already been successfully applied to treat patients in specialized hospitals, and many studies evaluated its efficacy on animal models too [40,41,42]. For what concerns clinical cases, MagForce AG [43], a company specialized in nanocancer therapy for treatment of tumors, initiated in 1997 a more than decennial study on materials and treatment methods, finally receiving the European Conformity (CE) marking for its MFH system in 2010. To date, it can boast the treatment of more than 100 patients affected by different types of cancer, e.g., glioblastoma multiforme and prostate cancer, spreading the MFH protocol to German, Polish and, recently, US hospitals. It is worth noting that in all successful cases (life expectation increased until 1.5 times), MFH is applied in combination with photon radiation therapy and, when proper, chemotherapy. The material injected intratumorally consists of aminosilane-coated magnetic nanoparticles (MNPs) with an iron-oxide magnetite core of a diameter d ≈ 12–15 nm and dispersed in an aqueous solution [44,45,46,47].

The patients underwent six semi-weekly hyperthermia sessions for 60 min: after the MNPs injection they were exposed to an alternating magnetic field (*f* = 100 kHz, µ_0_H = 2.5~19 mTesla) combined with radiotherapy [48]. At the preclinical level, for instance MNPs were used for a case of glioblastoma multiforme on Wistar rats, using µ_0_H = 20 mTesla and *f* = 874 kHz (40 min at T = 42 °C) [49]. Here, as in many other in-vitro and in-vivo cases, the Brezovich limit is exceeded, thus allowing the release of more thermal energy [50,51,52]. Remarkably, in most preclinical cases, superparamagnetic particles based on a Fe_3_O_4_ or γ-Fe_2_O_3_ core with d ≈ 20–22 nm are used, properly coated to prevent undesired aggregation and for ensuring biocompatibility [53,54].

In addition to X-ray radiotherapy plus MFH therapy, some cases of hyperthermia combined to HT have been recently reported. Datta et al. [55] report on two patients with unresectable soft-tissue sarcomas in the lower leg. They were treated with local hyperthermia (RF waves) once a week, in combination with a daily proton therapy (for 7 weeks), achieving functional limb preservation with nearly total tumor control. As a second example, Maeda et al. [56] suggested that hyperthermia (water bath at T = 42.5 °C for 1 h), applied immediately after radiation exposure, might induce hypersensitization to hadron radiation (protons and carbon ions). Thirdly, Ahmad et al. [57] collected preliminary data on the sensitization of cells to proton therapy using A549 lung cancer cells subjected to hyperthermia treatment (T = 42 °C using a heating pad) and to proton irradiation. Their results showed that the cell survival fraction dropped on average by 10–15% both at 2 and 4 Gy.

As concerns the combined action of radiotherapy, MNPs and hyperthermia, it should be noted that their effect could be synergistic or additive, as is usual in cancer treatments by means of, e.g., radiotherapy combined with chemotherapy, immunotherapy, surgery, hyperthermia and so on. For example, Dong et al. [58] show the synergistic osteosarcoma therapeutic strategy when hyperthermia is combined with elaborately catalytic Fenton reaction achieved by Fe_3_O_4_ and CaO_2_ NPs. On the other hand, Ito et al. [59] for the treatment of HER2-overexpressing cancer reported the combination of antibody therapy with magnetic hyperthermia showing an additive effect. Thus, being that killing tumor cells is the main goal, both additive and/or synergistic effects have been shown to be able to reduce the survival of tumor cells.

Although sparse data related to radiotherapy plus Hyp are available in the literature, there is a clear lack of systematic studies regarding the possible combination of HT and Hyp where MFH is used for locally increasing temperature. The present work aims to address the lack of investigation in this direction, by presenting a novel and promising approach directed to pancreatic BxPC3 tumor cells treatment. With the use of HT plus Hyp and, for comparison, of X-ray irradiation plus Hyp, we show that the clonogenic survival of BxPC3 cell cultures, decreased at first by HT, is further diminished by MNP uptake and Hyp treatment, the last one giving an additive killing effect of about 15–30% (MNPs action possibly being synergistic when photons are used). Moreover, when a 0.75 Gy carbon ions irradiation is used, we noted a significant increase in DNA double-strand-breaks at 6 h due to MNPs uptake and hyperthermia.

Our experimental results clearly remark the better efficacy of a dual-therapy treatment with respect to single therapy case and pave the way to translate the proposed protocol to clinic.

## 2. Materials and Methods

### 2.1. Synthesis and Characterization of Nanoparticles

All the samples were prepared under inert atmosphere using commercially available reagents. Benzyl ether (99%), oleic acid (OA, 90%), oleylamine (OAM, ≥98%), meso-2,3-Dimercaptosuccinic acid (DMSA), toluene (anhydrous, 99.8%), dimethyl sulfoxide (anhydrous, ≥99.9%) and sodium hydroxide (NaOH, ≥98%, pellets) were purchased from Aldrich Chemical Co. Iron(III) acetylacetonate 99% (Fe(acac)_3_) from Strem Chemicals Inc. Hydrochloric acid (HCl, ≥37%) and absolute ethanol (EtOH) were purchased from Fluka. Ultrapure water was obtained from a Milli-Q^®^ Synthesis system from Millipore, Temecula, CA, USA. All chemicals were used as received.

In brief, Fe(acac)_3_ (2.83 g, 8 mmol), OAM (8.56 g, 32 mmol), OA (9.04 g, 32 mmol) and benzyl ether (80 mL) were mixed and magnetically stirred under a flow of nitrogen in a 250 mL three-neck round-bottom flask for 15 min. The resulting mixture was heated to reflux (290 °C) at 25 °C/min and kept at this temperature for 90 min under a blanket of nitrogen and vigorous stirring. The black-brown mixture was cooled at room temperature and ethanol (60 mL) was added, causing the precipitation of a black powder. The product was magnetically separated with a permanent magnet, washed several times with ethanol and finally redispersed in toluene.

Afterwards, 400 mg were dispersed in toluene (60 mL), added to a solution of meso-2,3-dimercaptosuccinic acid DMSA (600 mg) in dimethyl sulfoxide (DMSO, 15 mL), sonicated for 1 h and finally incubated at room temperature for 12 h in a rotating agitator. The precipitate was magnetically separated with a permanent magnet, washed several times first with DMSO and then with ethanol and finally redispersed in MilliQ water (80 mL). The suspension was then basified to pH 10 with sodium hydroxide and adjusted to pH 7.4 with hydrochloric acid to make it stable.

The physical characteristics of the MNPs (e.g., size distribution and zeta potential characterized using a dynamic light scattering (DLS) instrument (Malvern Zetasizer ZS, Malvern Instruments Ltd., Malvern, UK). The morphology of the MNPs was determined using transmission electron microscopy, TEM (CM12 PHILIPS Transmission Electron Microscope, 100 kV).

Powder X-ray diffraction (XRD) measurements were carried out using a Bruker D8 Advance diffractometer equipped with a Cu Kα radiation (λ = 1.54178 Å) and operating in θ–θ Bragg Brentano geometry at 40 kV and 40 mA. Lattice parameters, a, and the mean crystallite diameters, d_XRD_, were evaluated using the TOPAS^®^ software (Bruker) using the method of the fundamental parameter approach considering a cubic space group Fd–3m. The surfactant percentage was determined by elemental analysis on carbon, hydrogen and nitrogen (CHN analysis) performed by a CHN-S Flash E1112 Thermofinnigan Elementary Analyzer.

Magnetic measurements were performed using a SQUID magnetometer (Quantum Design MPMS, San Diego, CA, USA) operating in the 2–350 K temperature range with applied fields up to 5 T. The powder sample was hosted in Teflon tape and then pressed in a pellet to prevent preferential orientation of the nanocrystallites under the magnetic field. The obtained values of magnetization were normalized by the weight of ferrite present in the sample and expressed in Am^2^/kg of ferrite.

### 2.2. Cell Culture

BxPC3 cells were obtained from ICLC (Interlab Cell Line Collection, Genova, Italy). Cells were maintained at 37 °C in a humidified atmosphere containing 5% CO_2_ in air as exponentially growing cultures in RPMI 1640 media (Roswell Park Memorial Institute, Sigma-Aldrich, St. Louis, MO, USA) supplemented with 10% fetal bovine serum (FBS, Sigma-Aldrich) and gentamicin (50 mg/mL; Sigma-Aldrich). In these conditions, the doubling time was 35 ± 2 h and the plating efficiency (PE) was about 50%.

### 2.3. Cell Toxicity

To assess the effect of MNPs on cell proliferation, BxPC3 cells in the logarithmic growth phase were treated with MNPs at a different concentration for 24 and 48 h of incubation. Cell toxicity was assessed by the clonogenic survival quantified after two weeks and was around 50–60% for cells incubated with MNPs of 50 μg/mL (for further data see Tables S1 and S2). Cell toxicity measured by the Trypan Blue assay after 48 h was around 3% for the same concentration. Results obtained from the cell cycle analysis of cells treated with MNPs (50 and 100 µg/mL) for 48 h have not shown alterations in the cell cycle phases (see Table S3).

### 2.4. Cellular Uptake of MNPs

To quantify the cellular uptake of MNPs (after 48 h of incubation at a concentration of 50 µg/mL), elemental iron (Fe) was quantitatively measured by inductively coupled plasma optical emission spectrometry (ICP-OES) with iCAP 6200 Duo upgrade, Thermofisher. Digestion with nitric acid was carried out at room temperature (T = 22 °C). The mean uptake (computed by averaging the results of all the ICP measurements performed for different experiments) was about 20 pg(Fe)/cell (see Table S4 for more details).

### 2.5. Irradiation

Cell irradiation with carbon ions was performed using the synchrotron-based clinical scanning beams (fixed horizontal beam line) at the Centro Nazionale di Adroterapia Oncologica (CNAO, Pavia). Since our cells must be placed in appropriate sample holders (that must respect the CNAO beamline geometry) and they deposit in the form of monolayers, to have a sufficient number of cells (1.5 × 10^6^) and irradiate them with the same dose we chose T25 flasks (25 cm^2^ surface). The flasks were placed vertically inside a water phantom put at the isocenter on the treatment table, at a depth of 15 cm, corresponding to the mid spread-out Bragg peak (SOBP). The SOBP (6 cm width, from 12 to 18 cm depth in water) was achieved with active beam energy modulation, using 31 different energies (246–312 MeV/u, Linear energy transfer (LET) of about 45 keV/μm). Samples were irradiated at different doses (0–2 Gy).

Photon beam irradiation of cell cultures (dose 0–7 Gy) was performed using a 6 MV linear accelerator (VARIAN Clinac 2100C, Varian Medical Systems, Palo Alto, CA, USA) at the Fondazione IRCCS Istituto Nazionale dei Tumori, Milano, Italy. The flasks containing the cells were irradiated using a vertical beam 20 × 20 cm^2^ field, placing them horizontally at the isocenter in a water phantom at 5 cm depth.

### 2.6. Clonogenic Assay

The cells were plated in T25 flasks (about 6 × 10^5^ cell/flask) and after two days some cell samples were incubated with MNPs (50 µg/mL) for 48 h at 37 °C. Afterwards, the cells were exposed to radiation types (photons and carbon ions) at different doses and hyperthermia treatment to the determination of cell survival. After irradiation the cells were detached from the flasks (using 0.25% trypsin_EDTA), counted and reseeded in five T25 flasks for each dose at a suitable cell concentration and incubated for 14 days. The cells were then fixed with ethanol and stained with 10% Giemsa solution and colonies consisting of more than 50 cells were scored as survivors. Surviving fractions relative to the untreated cell samples were determined.

### 2.7. Double Strand Breaks Studies

After irradiation and/or hyperthermic treatment a portion of the BxPC3 cells were seeded in slide flasks (Thermo Fischer Scientific, Waltham, MA, USA) with 3 mL fresh medium and collected 6 and 24 h later. Cells were fixed in 4% paraformaldehyde (PFA), permeabilized with 0.2% Triton X–100 and blocked at 37 °C in BSA 1% (w/v) dissolved in phosphate-buffered saline (PBS). Samples were then coimmunostained overnight (ON) at 4 °C, using a rabbit polyclonal anti–53BP1 antibody (Novus Biologicals, Littleton, CO, USA) in combination with a mouse monoclonal anti–γH2AX antibody (Millipore, Temecula, CA, USA). After washes in PBS/BSA 1% samples were incubated for 1 h at 37 °C in the secondary Alexa 546 anti-mouse and Alexa 488 anti-rabbit antibodies (Invitrogen, Life Technologies, Carlsbad, CA, USA). Finally, slides were washed in PBS/BSA 1%, counterstained with DAPI (Sigma-Aldrich, St. Louis, MO, USA) and mounted using the fluorescent mounting medium Vectashiled (Vector Laboratories, Burlingame, CA, USA). Images were acquired with an Axio-Imager.M1 fluorescent microscope (Zeiss, Jena, Germany) and analyzed using ISIS software (Metasystems, Milano, Italy). The frequency of both the DNA damage marker foci per cell were scored in 100 nuclei in four independent experiments.

### 2.8. Magnetic Fluid Hyperthermia Setup

Magnetic hyperthermia experiments were performed using a MagneTherm™ set-up by Nanotherics, working at 109.8 kHz and amplitude 19.5 mTesla. The temperature of the samples was measured using an Optocon™ optical fiber thermometer positioned at the centre of the sample placed inside an Eppendorf PCR Tube (also called mini-Eppendorf). The sample holder was optimized to accommodate two Eppendorf Tubes (volume 0.2 mL). In fact, the temperature was detected in a “twin-sentinel” sample, placed next to the sample used for the clonogenic assay, since it is necessary, for survival studies, to keep the latter sterile. It should be remarked that, due to the necessary heat insulation and coil geometry (inner diameter 44 mm), and thus to very limited space, we were forced to use Eppendorf tubes in place of much bigger T25 flasks (and thus a pelletization process that followed the irradiation, to transfer the samples from T25 flasks to mini-Eppendorf, see below).

### 2.9. Magnetic Hyperthermia Treatment

For the hyperthermia treatments the irradiated cells with MNPs were trypsinized, centrifuged (1500 rpm for 10 min), the cellular pellets (about 1.5 × 10^6^ cells in 0.1 mL medium) were transferred into 0.2 mL polypropylene mini-Eppendorf tubes and placed within the MagneTherm™ system. The pelletizing effect, obligatory step to switch from the irradiation configuration (cells in flasks) to that used for hyperthermic treatment (cells in mini-Eppendorf tubes), responsible of an ulterior stress for the cells was taken into account and separated from MFH action in the reported clonogenic survival curves (see paragraph 3.2). This stress is due to all the operations (trypsinization, centrifugation and vortexing) necessary to transfer the irradiated cells from the T25 flask to the mini-Eppendorf. This stress is quantified by a reduced plating efficiency caused by cells that are damaged and therefore no longer proliferate or proliferate less; this effect was taken into account in renormalizing the clonogenic survival curves. When the evaluation of survival rate is given, the eventual stress effect was previously subtracted. A custom thermalization system, based on a Lauda Alpha A thermostat, and a polystyrene sample holder, was placed inside the MagneTherm™ coil to stabilize the initial temperature of the sample to the physiological value of 37 °C, to center the sample in the homogeneity region of the field and to minimize heat dissipation. An AMF (amplitude µ_0_H = 19.5 mTesla and frequency *f* = 109.8 kHz) was applied immediately after the irradiation to increase the temperature of the cells up to 42 °C; this temperature was later maintained for 30 min by changing the temperature of the water circulating within the thermalization system. The field and frequency values have been selected not to exceed Brezovich’s criterion excessively, as these values reflect those used in the clinic [43].

## 3. Results and Discussion

### 3.1. Synthesis and Characterization of MNPs

Since experiments lasted more than 3 years, it was not possible to use the same batch of MNPs. Different batches of MNPs with similar features were then prepared following the same procedure. The characteristics of a representative sample are described in the following. The samples consisted of a spherical Fe_3_O_4_ core coated with meso-2,3-dimercaptosuccinic acid. The MNPs were synthesized by thermal decomposition of iron acetylacetonate (Fe(acac)_3_), in benzyl ether in the presence of oleic acid (OA) and oleylamine (OAM) as surfactants.

**Figure 1:**
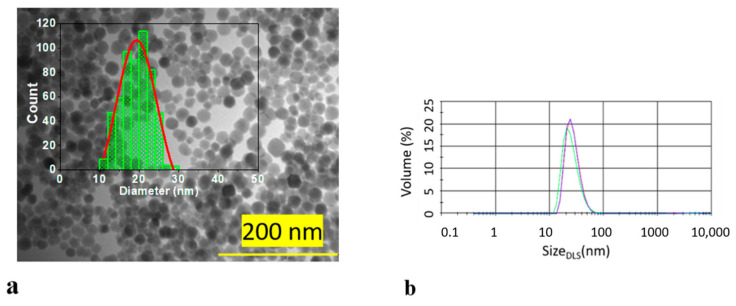
(**a**) Representative bright-field TEM image, together with the histogram of the size distribution (d_TEM_ = 19.2 ± 3.6 nm). The solid red line represents the best Gaussian fit. (**b**) Dynamic light scattering (DLS) diameter d_DLS_ measurements at pH 7.4. The green, blue and red lines represent data obtained from three different measurements.

The core morphodimensional characteristics were studied by means of transmission electron microscopy (TEM), using a CM12 PHILIPS transmission electron microscope. In Figure 1a we report a representative TEM image, which demonstrates the almost spherical shape of the particles; a mean core diameter value of d_TEM_ = 19.2 ± 3.6 nm was extracted from the histogram of the size distribution.

The X-ray diffraction pattern (see Supplementary Materials Figure S1) shows a single crystalline phase with a diffraction pattern compatible with the inverse spinel cubic structure characteristic of magnetite. The peaks all match the reference pattern (JCPDS 19–0629) as regards both position and intensity. The lattice parameter (a = 8.387 Å) is close to the one expected for magnetite (8.396 Å), suggesting a low degree of surface oxidation of the MNPs, which reasonably consist of a magnetite core surrounded by a thin maghemite shell. The crystallite diameter obtained by the Scherrer analysis (d_XRD_ = 18.7 ± 0.4 nm) is comparable to the one obtained by TEM measurement, suggesting that the MNPs are single crystals and present a high degree of crystallinity.

The MNPs were dispersed in pH 7.4 water after coating with DMSA (ca. 2% *w/w* as evaluated by CHN analysis). The successful DMSA functionalization and the stability in water solution were also confirmed by dynamic light scattering (DLS, Figure 1b) and zeta potential (Figure S2) measurements, which provided d_DLS_ = 27 ± 8 nm and Z = −30.5 ± 7.5 mV at pH 7.4.

The hyperthermic efficiency was estimated by evaluating the specific absorption rate (SAR). The SAR of the samples was calculated using the following formula: [60]

(1) $$SAR=\frac{m_{H_{2}O}\;c_{H_{2}O}+m_{Fe_{3}O_{4}}\;c_{Fe_{3}O_{4}}}{m_{Fe_{3}O_{4}}}\;\cdot \frac{\Delta T}{\Delta t}$$

where $c_{H_{2}O}=$ 4.18 J K^−1^ g^−1^ and $c_{Fe_{3}O_{4}}$= 0.62 J K^−1^ g^−1^ are the specific heat of water and magnetite in colloidal solutions, while *m*_*H*2*O*_ and *m*_*Fe*3*O*4_ are the respective masses. We neglected the contribution of the DMSA coating because of its small mass fraction. From the experimental temperature kinetics curve, *T* vs *t*, the SAR was estimated to be 110 ± 30 W/g_Fe3O4_ under an AMF of frequency *f* = 109.8 kHz and amplitude µ_0_H = 19.5 mTesla (for more details see Figure S3).

**Figure 2:**
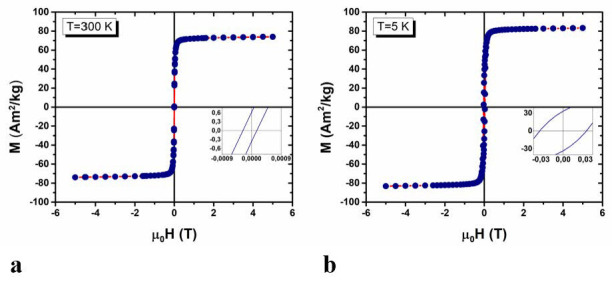
(**a**) Hysteresis loop measured at 300 K and (**b**) 5 K, in the field range ± 5 Tesla. In the insets, we report the low field regions.

The field dependence of the sample magnetization was investigated by means of SQUID magnetometry. In Figure 2 we reported the M vs. H curves obtained at 5 K and 300 K.

At T = 5 K MNPs showed hysteretic behavior with a coercive field µ_0_Hc = 30 ± 2 mTesla, while a negligible opening-up of the hysteresis loop was observed at T = 300 K (less than 0.3 mTesla, comparable to the remanent field). These results indicate that MNPs display a superparamagnetic behavior, as expected for magnetite based MNPs of this size. At both temperatures, the saturation magnetization, Ms, was very high (83 Am^2^/kg at 5 K and 74 Am^2^/kg at 300 K) and close to the bulk values (91.7 Am^2^/kg_Fe3O4_ at room temperature [61]), confirming the high crystallinity of the inorganic core.

### 3.2. Experimental Treatment Protocol

**Scheme 1:**
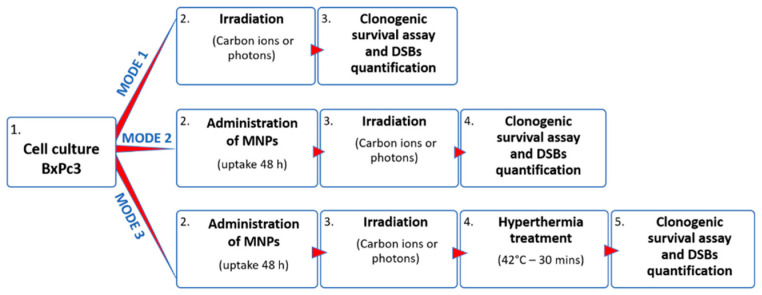
Description of the 3 different treatment modes used during the experiments. Clonogenic survival has been determined after simple irradiation (mode 1), magnetic nanoparticles (MNPs) administration and irradiation (mode 2) and MNPs administration plus irradiation and subsequent hyperthermia (Hyp; mode 3). DSB stands for a break in double-stranded DNA in which both strands have been cleaved.

As a novel cancer treatment, we investigated the possible synergistic/additive action of hadron therapy and hyperthermia on pancreatic cancer BxPC3 cell culture. The general experimental protocol, sketched in Scheme 1, comprised of three different treatment modes, indicated as 1, 2 and 3 (see next paragraph and Materials and Methods for more details).

The irradiation of samples (cells incubating or not MNPs) have been performed by a C ions beam at the synchrotron-based facility at the National Center for Oncological Hadron Therapy (CNAO) in Pavia, or by means of photons, using a 6 MV linear accelerator at the Fondazione IRCCS Istituto Nazionale dei Tumori in Milano (INT).

The MNPs, used to perform part of the hyperthermic process via MFH, have been administered to human pancreatic adenocarcinoma BxPC3 cells with a concentration 50 μg/mL in the culture medium. The cells were incubated for 48 h to favor cells’ uptake (see Materials and Methods) and thus improve the MFH efficacy.

**Figure 3:**
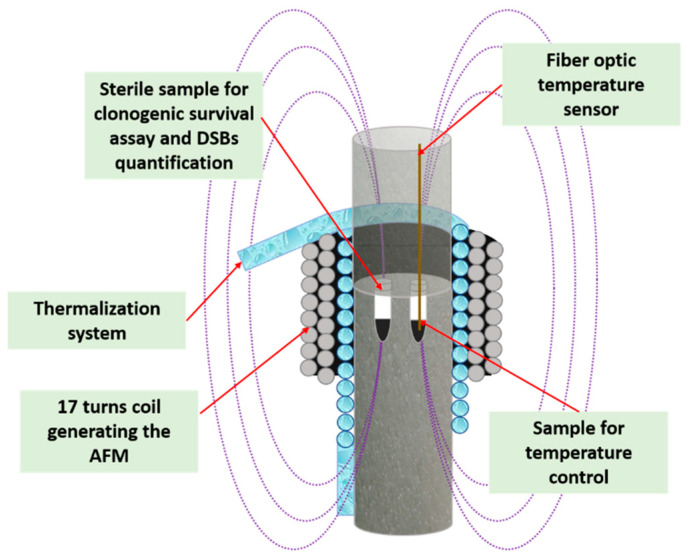
Representation of the hyperthermia setup. A thermalization system (bath heating) circulates water around the two sample vials, one sterile for the clonogenic survival assay and DSBs detection, and one for the temperature registration by a fiber optic probe.

In the cases of combined therapy (mode 3 in Scheme 1), after the irradiation, we applied Hyp to samples of cells incubating MNPs. The temperature was increased from 37 °C to 42 °C (ΔT = 5 °C) and then kept constant for 30 min, by means of bath heating (through a thermalization system that circulates water around the sample) and MFH, realized by an AMF of 19.5 mTesla amplitude and 109.8 kHz frequency. A sketch of the hyperthermia setup is shown in Figure 3. The amount of uptaken MNPs was estimated by inductively coupled plasma-optical emission spectrometry (ICP-OES) and found on average ~20 pg(Fe)/cell, calculated over different experiments. With this amount, on average about 40% of the heating is due to the application of the magnetic field, while the remaining temperature increase was obtained due to the thermalization system. It should be noted that, due to the small cell volume, the value of 20 pg(Fe)/cell corresponded to filling mostly the inner part and the surface of the cell and, thus, a quantity of heat release near to the highest one.

The biological effect of the different treatment modes was finally assessed:

(i) By using a clonogenic assay after two weeks. The clonogenic cell survival assay, i.e., the ability of a cell to produce a viable colony containing at least 50 cells, is considered as a gold standard method for studying cellular sensitivity to irradiation [62];
(ii) By estimating the number of non-repairable double strand breaks (DSBs) per cell after 6 and 24 h, through detection and counting of persistent repair foci, which are DSBs markers. This study was limited to carbon ions irradiation.

### 3.3. Clonogenic Survival Studies

#### 3.3.1. Carbon Ion Irradiation Experiments

**Figure 4:**
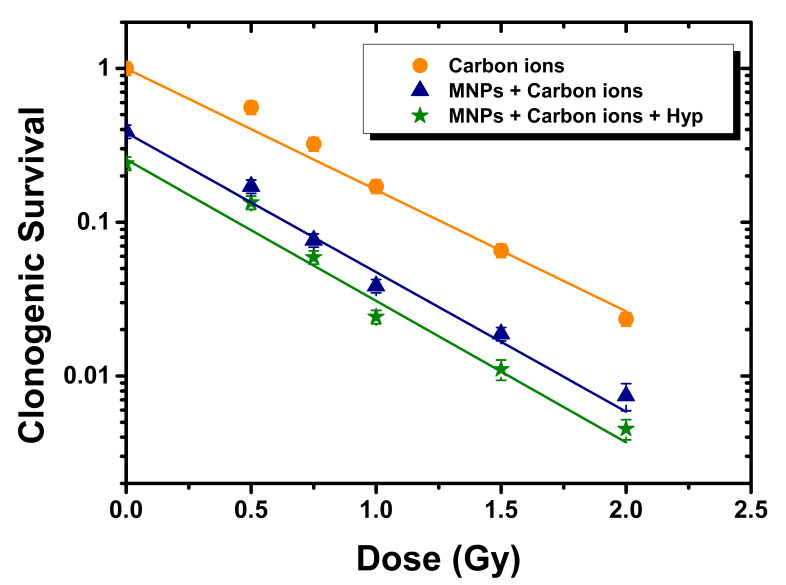
Clonogenic survival of BxPC3 cells culture for 3 different protocols (see text): hadron therapy (HT) only (orange circles), HT + MNPs administration (navy triangles) and HT + MNPs administration + Hyp (green stars). An additive effect of MNPs and Hyp is noted. Solid lines represent the best fit of the clonogenic survival (CS) curves as a function of the radiation dose D according to the law CS ∝ exp(–αD). From the fits: α_C ions_ = 1.82 ± 0.06 Gy^−1^; α_MNPs+C ions_ = 2.09 ± 0.15 Gy^−1^ and α_MNPs+C ions+Hyp_ = 2.11 ± 0.20 Gy^−1^.

At first, the assessment of the effect of the combination of HT + MNPs + Hyp was performed using the clonogenic assay 2 weeks after the experiment. Figure 4 shows survival data of BxPC3 cells treated with three different modes as described in Scheme 1: (1) only carbon ion (0–2 Gy) irradiation (HT, orange circles); (2) carbon ion irradiation after the administration of magnetic nanoparticles (HT + MNP, navy triangles) and (3) carbon ion irradiation on culture cells containing MNPs combined with the hyperthermia treatment for 30 min at 42 °C (HT + MNP + Hyp, green stars).

The results for each protocol were averaged over four independent experiments.

The following observations were found:

(i) At 0 Gy dose, i.e., unirradiated samples, clonogenic survival (CS) decreased from 1 to 0.4 ± 0.04 when MNPs were added, due to a MNP toxicity at 15 days. A further decrease of CS to 0.24 ± 0.02 was observed when also Hyp was applied (mode 3);
(ii) If carbon ions irradiation alone was applied, the CS decreased on increasing the dose, according to the law: CS ∝ exp(–αD) where D is the dose;
(iii) At all doses, once MNPs were added and irradiation was performed (mode 2), a decrease of CS with respect to irradiation only (mode 1) was observed;
(iv) At all doses, once Hyp was further added (mode 3), the CS dropped further.

It is thus straightforward to conclude that: (i) there is a sizeable MNP toxicity that could result in a potential additive therapeutic effect (although not synergistic) and (ii) hyperthermia grants an additional killing effect on tumor cells with respect to hadron therapy alone.

#### 3.3.2. Photon Irradiation Experiments

**Figure 5:**
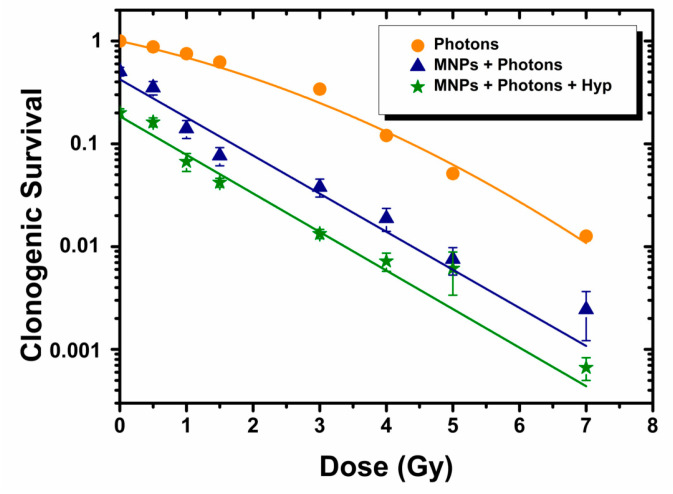
Clonogenic survival of BxPC3 cells incubated with or without MNPs, irradiated with photons (0–7 Gy) and combined (or not) with Hyp. Solid lines represent the best fit of the clonogenic survival (CS) curve as a function of the radiation dose D according to the law CS ∝ exp(–αD-βD^2^) for photons alone, and to CS ∝ exp(–αD) for the other two treatments. From the fit: α_Photons_ = 0.32 ± 0.06 Gy^−1^; β_Photons_ = 0.05 ± 0.01 Gy^−2^; α_MNPs+Photons_ = 0.85 ± 0.09 Gy^−1^ and α_MNPs+Photons+Hyp_ = 0.86 ± 0.06 Gy^−1^.

To compare the effect of HT plus Hyp treatment with a more conventional one making use of photon irradiation plus Hyp, we performed experiments by using a linear particle accelerator Linac at INT. Figure 5 shows survival data of BxPC3 cells exposed to different doses of 6 MV photons (0–7 Gy) and to Hyp at 42 °C for 30 min, as averaged over two independent experiments.

In a similar way to the CNAO experiments, three treatment modes were used: (i) photon irradiation alone (orange circles), (ii) photon irradiation after the administration of MNPs for 48 h (navy triangles) and (iii) photon irradiation on cells incubating MNPs combined to 30 min at 42 °C Hyp treatment (green stars).

One could observe the following:

(i) As in the HT case, at 0 Gy dose, i.e., unirradiated samples, clonogenic survival (CS) decreased from 1 to 0.5 ± 0.05 when MNPs were added, and a further decrease of CS to 0.2 ± 0.02 was observed when also Hyp was applied (mode 3);
(ii) If only photon irradiation was applied, the CS decreased once the dose was increased according to the linear quadratic model: CS ∝ exp(–αD–βD^2^);
(iii) At all doses, CS decreased once MNPs were added with respect to irradiation with photons only, confirming the results found for HT;
(iv) When MNPs were added, the CS vs. D model changed to CS ∝ exp(–αD), which corresponded to a modification of the cells response to photon irradiation; thus the typical shoulder of the dose–survival curves, found after treatment with radiation alone, was removed;
(v) As in HT, once Hyp was further added, the CS dropped further; also in this case, the dose–survival curve obeyed the law CS ∝ exp(–αD).

Therefore, also in this case we could conclude that: (i) there was a MNP toxicity, comparable to the HT case, and (ii) Hyp gave an additive killing effect on tumor cells with respect to photon irradiation alone.

Let us compare shortly the results of carbon ions and photon irradiation alone. As already observed for other type of tumors [63,64,65] and other pancreatic tumor cell lines [51], also in the case of BxPC3 cells the survival fractions obtained when carbon ion radiotherapy was used were lower than those for the photon-irradiated samples. This means that carbon ion radiotherapy shows an enhanced efficacy as compared to standard photon radiotherapy. Moreover, as mentioned in the introduction, due to the physical characteristics of high-LET particle radiation (carbon ions) the maximum dose deposition occurs at the so-called Bragg Peak, the depth of which can be adjusted at will within the target tissue. Based on this custom dose profile it should be possible to more accurately administer maximum doses to the tumor with minimum adverse effects to the surrounding tissues. For carbon ion and photon irradiations α values were determined and are given in the captions of Figure 4 and Figure 5. As expected, α values were significantly higher for carbon ions than for photons, reflecting a steeper decline of the initial slope of the survival curves for high-LET beams.

Remarkably, particle radiation has been proven to have a higher relative biological effectiveness (RBE) achieving an increased cytotoxic effect when compared with conventional photon therapy. Additionally, we determined the carbon ions RBE by comparing cell survival data after carbon and photon irradiations as the ratio between the dose of reference radiation (6 MeV photon) and the carbon ions are necessary to produce the same biological effect.

At 10% of the clonogenic survival, the RBE of the CNAO carbon ions beam was equal to about 3.5. This result suggests that the use of hadrons in cancer therapy for the treatment of pancreatic tumors could be a promising modality. Until now the carbon ions RBE values reported in the literature are few, but they seem to agree with our result. El Shafie et al. [66] have found that the RBE of carbon ion irradiation for the BxPC3 cell line ranged from 1.5 to 3.5, depending on the survival level and dose, and data published by Oonishi and colleagues confirms, for the same cell line, the increased RBE of carbon ions irradiation [67].

Remarkably, as the MNPs presence increased the cell mortality rate and, in the case of photons, changed the dose-survival response law, we guess that they have a radiosensitizing (possibly synergistic) effect on BxPC3 cells. Similar results were obtained by Li et al. [68], Liu et al. [69] and Wang et al. [70] with gold nanoparticles and X-rays on 4T1, EMT-6 murine breast carcinoma and HeLa cells, respectively. In another perspective, Goel et al. [71] report progress in nanoparticles-mediated radiosensitization.

### 3.4. Double Strand Breaks Studies for HT

As a measure of the single/combined effect of hyperthermia and carbon ions irradiations in the induction of DNA damage, the kinetics of DNA double strand breaks rejoining has been evaluated by means of γH2AX and 53BP1 foci formation by immunofluorescence analysis. Both γH2AX (phosphorylation at Ser-139) and 53BP1 are well validated markers of DNA double-strand breaks. [72,73].

**Figure 6:**
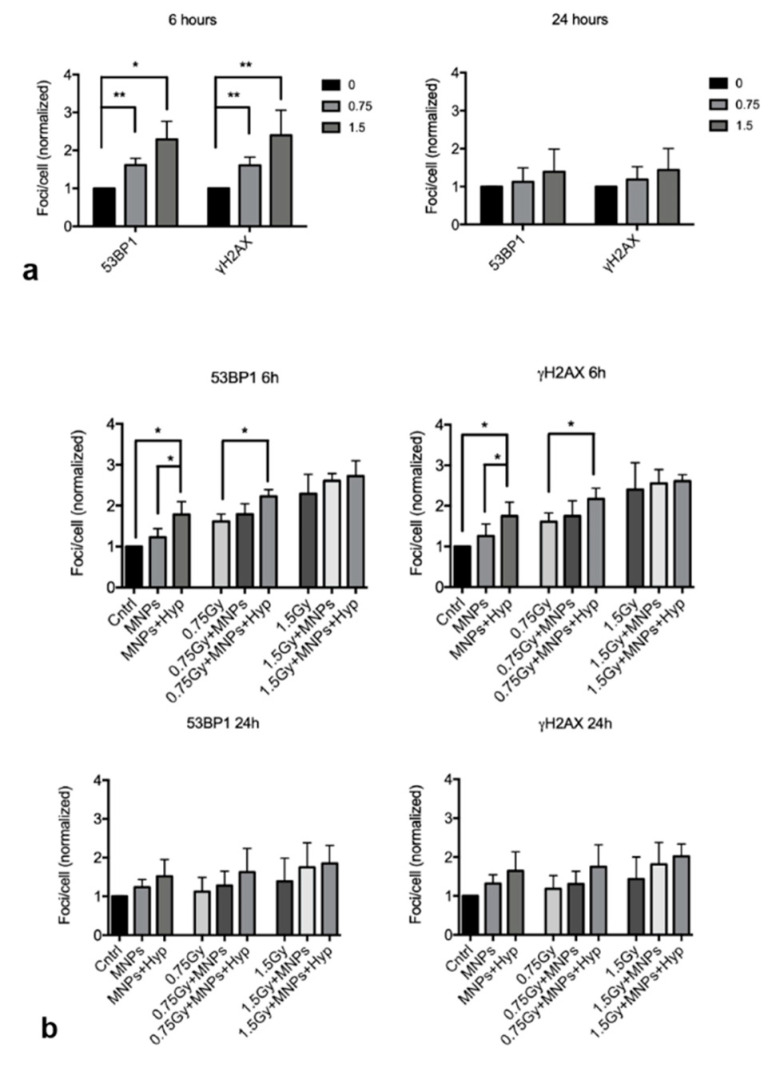
Analysis of 53BP1 and γH2AX foci induction after 6 and 24 h from the exposure to 0.75 and 1.5 Gy of carbon ions alone (**a**) and in combination with MNP uptake and/or Hyp in BxPC3 pancreatic tumor cells (**b**). * indicates *p* < 0.05, ** indicates *p* < 0.01 (one-way ANOVA and Tukey’s multiple comparison post-test).

Results from four experiments carried out at the CNAO facility were collected and analyzed after exposure of BxPC3 pancreatic tumor cells to 0.75 and 1.5 Gy and harvested at 6 and 24 h from the three different treatment modes. In Figure 6 we report the normalized “foci/cell” values at different doses. It is observed: (i) as expected carbon ions radiation alone (mode 1) increased the number of DSBs with respect to control, i.e., untreated, samples (control, Figure 6a); such a difference was more pronounced at 6 h from treatment, indicating that at 24 h cell repair is more efficient for both DSBs markers used; (ii) at a zero dose, hyperthermia treatment (mode 3) further increased the number of DSBs at 6 h compared to samples without Hyp (mode 1 and 2 treatments, Figure 6b); (iii) at 0.75 Gy, Hyp induced a significant increase in DSBs after 6 h, for both γH2AX and 53BP1 foci, while at 1.5 Gy the differences in the DSBs number between the three different treatment modes was minimal and (iv) at 24 h there were lower DSB values for both doses and for all treatment modes.

These results were similar to those reported by Ma et al. [74] and suggest that the MNPs+Hyp has a radiosensitization effect and inhibits cellular DNA repair mechanisms.

Although the available literature supports the notion that the hyperthermic effect is dependent on the LET of ionizing radiation, the potential molecular mechanism underlying hyperthermia radiosensitization for particle radiation still remain scantly investigated and poorly known. As for low-LET radiations, a major contribution in the untangling of such a mechanism/s was provided by using Chinese hamster ovary (CHO) cell lines specifically defective in the two processes devoted to the repair of radiation-induced DNA DSBs, and namely non homologous end joining (NHEJ) and homologous recombination (HR). In this respect, exposing CHO wild-type cells and a panel of repair defective counterparts to protons (LET = 1 keV/μm 42.5 °C water bath, 1 h) and carbon ions (LET = 13–70 keV/μm; 42.5 °C water bath, 1 h) it was demonstrated a prevalent contribution of HR over NHEJ in radiosensitization, probably affecting the processing of a subset of DNA DSBs lesions [56]. Interestingly, at the molecular level it was shown that Hyp (T > 41 °C) did inhibit HR in human and mouse cells, affecting some of the key players in this process (e.g., delaying recruitment of RAD51 at radiation-induced foci, degrading BRCA2, inactivating RPA, reducing the level of MRN complex, etc.; see [75]). In addition, since heat may explicate the pleiotropic effect on cells, it is generally thought that cytotoxic or sensitizing effects of Hyp cannot be attributed to deactivation of a single DNA repair mechanism, but rather to influencing many pathways (e.g., cell cycle progression and activation of checkpoints) on multiple levels [75,76].

## 4. Conclusions

In this work, we reported on a combination of carbon ion therapy and hyperthermia applied to pancreatic adenocarcinoma cells BxPC3, for assessing its antitumor efficacy and proposing protocols for future clinical applications. The results obtained were compared to those achieved with a 6 MV photon beam irradiation joint to hyperthermia. A significant part of the heating power was provided through MFH (40% of the total heating), assisted by magnetite nanoparticles with a core diameter of 19 nm and coated with an organic biocompatible ligand, namely DMSA. The hadron carbon ion therapy was shown to have RBE ~3.5, thus confirming a greater efficacy with respect to photon therapy. The clonogenic survival results with respect to a simple irradiation of culture cells, clearly show: (i) at all HT/photon irradiation doses, a further killing (toxicity) additive effect of about 50–60% due to the MNPs cellular uptake and (ii) a significant killing effect of hyperthermia, consisting in an additive 15–30% of total CS, for both irradiation protocols (MNPs action possibly being synergistic when radiation therapy is delivered by photons). Moreover, a significant increase of DNA-DSBs was observed at 6 h after 0.75 Gy-dose HT irradiation plus administered MNPs and Hyp, with respect to the sample exposed to irradiation only.

The increased efficacy of hadron therapy combined with hyperthermia (applied immediately after) lays the foundations for future preclinical studies. Furthermore, these encouraging results point in the direction of further investigating this combination, with a view to finally translate it to a clinical application.

## Supplementary Materials

The following are available online at https://www.mdpi.com/2079-4991/10/10/1919/s1, Figure S1: XRD pattern, Figure S2: Zeta potential measurements, Figure S3: SAR estimation method, Tables S1 and S2: determination of optimal time of MNPs uptake and concentration, Table S3: BxPC3 cell cycle analysis after 48 h of treatment with MNPs, Table S4: cellular uptake of MNPs evaluation, Figure S4: dissolution experiments by the radiotracing method.
