# Supplementary material for: Hadron Therapy, Magnetic Nanoparticles and Hyperthermia: A Promising Combined Tool for Pancreatic Cancer Treatment
Source: Nanomaterials (Basel). 2020 Sep 25;10(10):1919. doi: 10.3390/nano10101919 (PMC7600442; doi:10.3390/nano10101919)
Supplement: Supplementary file 1 [file nanomaterials-10-01919-s001.pdf]

Supporting information

Hadron Therapy, magnetic nanoparticles and Hyperthermia: a promising combined tool for pancreatic cancer treatment

Francesca Brero [1,*] Martin Albino [2], Antonio Antoccia [3], Paolo Arosio [4], Matteo Avolio [1], Francesco Berardinelli [3], Daniela Bettega [4], Paola Calzolari [4], Mario Ciocca [5], Maurizio Corti [1], Angelica Facoetti [5], Salvatore Gallo [4], Flavia Groppi [6], Andrea Guerrini [2], Claudia Innocenti [2,7], Cristina Lenardi [4,8], Silvia Locarno [4], Simone Manenti [6], Renato Marchesini [4], Manuel Mariani [1], Francesco Orsini [4], Emanuele Pignoli [9], Claudio Sangregorio [2,7,10], Ivan Veronese [4], Alessandro Lascialfari [1,*]

1 Dipartimento di Fisica and INFN, Università degli Studi di Pavia, Pavia 27100, Italy
2 Dipartimento di Chimica, Università di Firenze and INSTM, Sesto Fiorentino (FI) 50019, Italy
3 Dipartimento di Scienze and INFN, Università Roma Tre, Roma 00146, Italy
4 Dipartimento di Fisica and INFN, Università degli Studi di Milano, Milano 20133, Italy
5 Fondazione CNAO, Pavia 27100, Italy
6 Dipartimento di Fisica, Università degli Studi di Milano and INFN, Lab. LASA, Segrate (MI) 20090, Italy
7 ICCOM-CNR, Sesto Fiorentino (FI) 50019, Italy
8 C.I.Ma.I.Na., Centro Interdisciplinare Materiali e Interfacce Nanostrutturati, Milano 20133, Italy
9 Fondazione IRCSS Istituto Nazionale dei tumori, Milano 20133, Italy
10 INFN, Sezione di Firenze, Sesto Fiorentino (FI) 50019, Italy
* Correspondence: alessandro.lascialfari@unipv.it (A.L); francesca.brero01@universitadipavia.it (F.B); Tel.: +39-0382-987-483

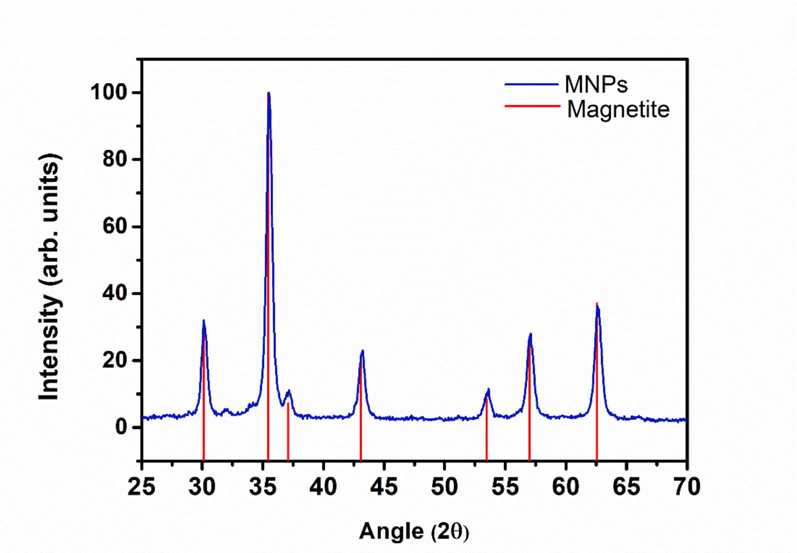

Figure S1. XRD patterns, compared to the reference pattern (red vertical bars) of magnetite (JCPDS 19-0629).

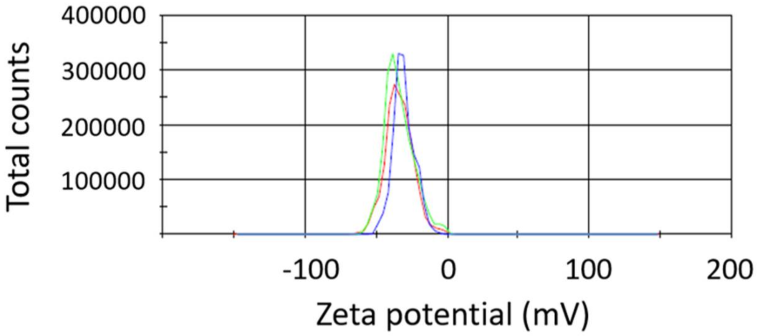

Figure S2. Zeta potential measurements at pH 7.4. The green, blue and red lines represent data obtained from three different measurements. The zeta potential measurements show that the surface charge of the MNPs is negative, (Z_{pot} = -30.5 ± 7.5 mV at pH 7.4). The negative surface charge is attributed to the carboxyl groups of the DMSA molecules and the high zeta potential value justifies the stability of the sample in water.

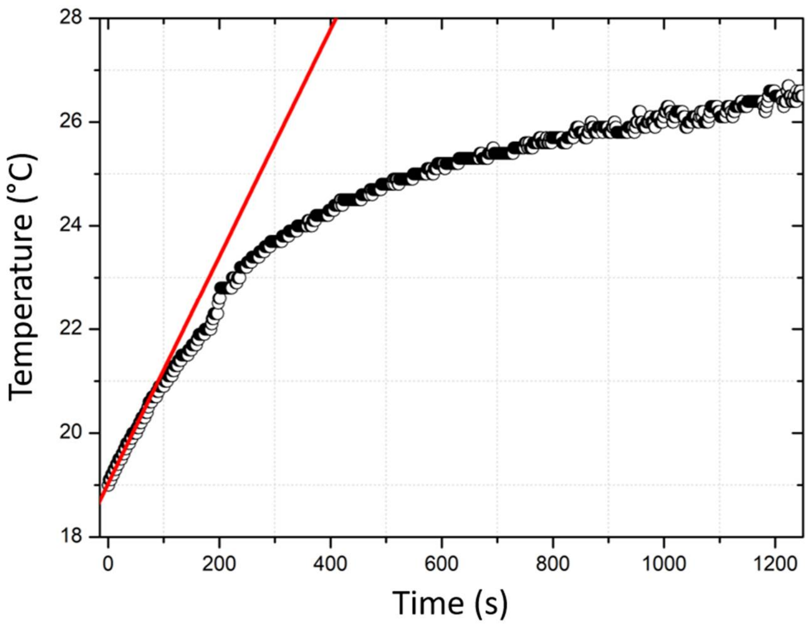

Figure S3. Temperature kinetics (circles) of water suspensions of MNPs as a function of application time interval (Δt) of the alternating magnetic field (amplitude μ_0H=19.5 mTesla and frequency *f*=109.8 kHz). The red line is a fit to the temperature vs. time curve for Δt→0 (red lines): the SAR was estimated by the so-called initial-slope method, evaluating the rate of temperature increase (from the initial temperature T_0) by a linear fit on a short time range just after the application of the field. At short times, the effect of thermal dissipation can be neglected, and the thermal inertia of the system is taken into account by starting the fit a few seconds (5–20 s) after the application of μ_0H. The ΔT/Δt ratio for t→0 was measured directly from the curve.

Concentration μg/mL	Uptake	Survival	$\sigma_{survival}$
0	24 h	1.00	0.10
	48 h	1.00	0.10
50	24 h	0.42	0.04
	48 h	0.40	0.04
100	24 h	0.38	0.04
	48 h	0.38	0.04

Table S1. Results of experiments performed to determine the optimal time of uptake and the concentration of MNPs in the cell culture medium are shown. As it can be observed in Table S1, the cells survival obtained for the samples with uptake of the nanoparticles for 24 hours and for 48 hours are almost the same. Moreover, the concentration of 50 μg/mL seems to cause the same cytotoxicity of the concentration 100 μg/mL.

Concentration μg/mL	Uptake	Survival	$\sigma_{survival}$
0	24 h	1.00	0.10
10	24 h	0.88	0.09
25	24 h	0.68	0.07
50	24 h	0.41	0.04
100	24 h	0.38	0.04

Table S2. Results of experiments performed to determine the optimal concentration of MNPs in the cell culture medium. As it can be observed in Table S2, the cell survival (after 15 days from the uptake process) drops increasing the MNPs concentration, reaching a plateau for c > 50 ug/mL. Indeed, the concentration of 50 μg/mL and of 100 μg/mL seems to cause the same cytotoxicity effects, probably due to an uptake upper limit due to the small cells internal volume. Finally, we used c=50 μg/mL because with lower c the amount of uptaken MNPs is too low to allow a magnetic hyperthermia treatment, and for c > 50 μg/mL MFH efficacy does not increase.

Sample	G1 phase	S Phase	G2/M Phase	Apoptosi
Control	59.83%	25.4%	13.77%	/
MNPs – 50 μg/ml	57.11%	21.54%	13.25%	8%
MNPs – 100 μg/ml	56.9%	21.43%	13.13%	8.5%

Table S3. Cell cycle analysis of BxPC3 cells measured by flow cytometry after 48 h of treatment with MNP (50 and 100 μg/mL)

Cell cycle distribution was measured after 48 h of incubation with MNPs at concentration of 50 and 100 μg/mL. For cytofluorimetric analysis the cell samples were treated with Ribonuclease A (100 μg/mL—Sigma Aldrich) for 30′ at 37 °C and then stained with propidium iodide (50 μg/mL—Sigma Aldrich) for about 12–14 hours. The flow cytometer BD ACCURI C6 was used for the experiments and at least 20,000 cells were counted at each point. The proportion of cells at different phases was gated and calculated using the ModFit Lt software.

There were no significant variations in the values of the different cell phases for all samples, treated with MNPs for 48 hrs and not treated (control). Similar results have been found in several works such as: Ma et al.[1] and Calero et al.[2], who conclude that MNPs incorporated into cells does not alter the cell cycle. Note instead a certain percentage of apoptotic cells in the samples were treated with MNPs due to the cytotoxicity of the nanoparticles.

Experiment	Cellular Uptake $pg_{(Fe)}$/cell
Experiment 1	16.6
Experiment 2	25.6
Experiment 3	17.9

Table S4. Mean cellular uptake (results of ICP measurements).

Dissolution experiments by the radiotracing method.

A dissolution test on Fe_3O_4 nanoparticles (Fe_3O_4-MNPs) in the culture medium (RPMI 1640 media) supplemented with 10% fetal bovine and gentamicin (50 mg/mL) was carried out by the radiotracing method.

Radiolabeled [^{59}Fe] $^{59}Fe_3O_4$-MNPs were prepared at the Triga Mark II Nuclear Reactor of the LENA Laboratory (University of Pavia). The radioactivity measurements were carried out by X-ray spectrometry using a high purity germanium (HPGe) detector (EG&G Ortec, 15% relative efficiency, FWHM = 2.2 keV at 1.33 MeV). Within the experimental errors, the concentration of the irradiated Fe_3O_4-MNPs was in good agreement with the expected nominal one. The $^{59}Fe_3O_4$-MNPs were resuspended in 10 mL of Millipore H_2O and submitted to the dissolution test by incubating the MNPs in the culture medium at a final concentration of 150 µg mL^{-1} for 24, 48 and 72 h at 37 °C.[3] After incubation the mixtures were centrifuged at 2000*g* for 10 min and the fraction of ^{59}Fe in the supernatants (“ionic Fe”) measured by radioactive counting. The kinetic of the ^{59}Fe released by in culture medium (Figure S4) shows concentrations of “ionic Fe” ranging from 1.25% to 3.3% of MNPs mass, suggesting that under our experimental conditions only a small dissolution of $^{59}Fe^3O_4$-MNPs in culture media occurred.

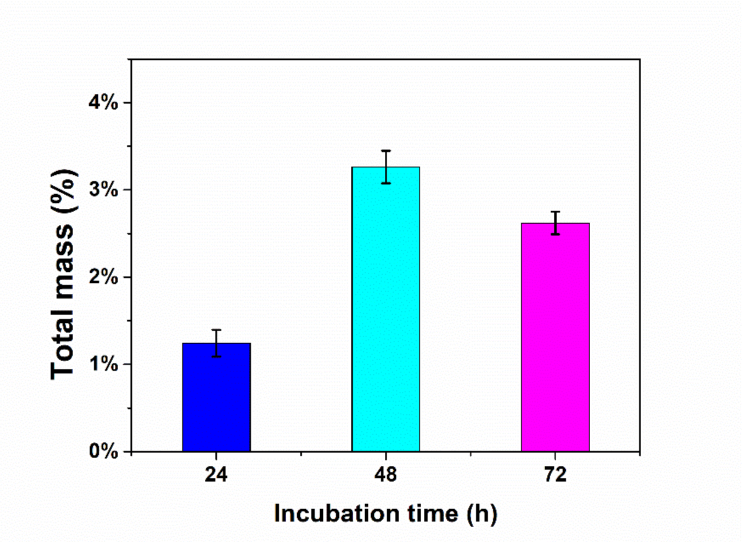

Figure S4. Timed dissolution of $^{59}Fe_3O_4$-MNPs in culture medium. Data are expressed as % of the initial mass of MNPs. Means of three measurements. Bars represent standard errors.

References

[1] J. Ma, Z. Zhang, Z. Zhang, J. Huang, Y. Qin, X. Li, H. Liu, K. Yang, G. Wu, *Int. J. Hyperth.* **2015**, 31, 800.

[2] M. Calero, M. Chiappi, A. Lazaro-Carrillo, M. J. Rodríguez, F. J. Chichón, K. Crosbie-Staunton, A. Prina-Mello, Y. Volkov, A. Villanueva, J. L. Carrascosa, *J. Nanobiotechnology* **2015**, 13, 1.

[3] R. Gornati, E. Pedretti, F. Rossi, F. Cappellini, M. Zanella, I. Olivato, E. Sabbioni, G. Bernardini, *J. Appl. Toxicol.* **2016**, 36, 386.
